# Psychometric analysis of the revised CompLEC test to measure reading speed and reading comprehension in university students

**DOI:** 10.1186/s40359-023-01374-1

**Published:** 2023-10-12

**Authors:** Claudia Milagros Arispe Alburqueque, Oriana Rivera-Lozada, Judith Soledad Yangali Vicente, Melba Rita Vásquez Tomás, Lindsey W. Vilca, Ramiro Alcides Enciso Soto, María Ysabel Alvarez Huari

**Affiliations:** South American Center for Education and Research in Public Health, Universidad Norbert Wiener, Lima, Perú

**Keywords:** Reading comprehension, Reading speed, University student, Psychometric properties

## Abstract

**Background:**

At university level, reading comprehension is one of the most important linguistic competences in the professional training of students because it is an instrument that enables the acquisition and production of scientific knowledge. Likewise, at this level of education, speed reading becomes a technique that allows to make the most of the time devoted to reading and to develop the ability to concentrate. However, there are not many instruments in the scientific literature that measure these two variables; therefore, the objective of this study was: To determine the psychometric properties of the revised compLEC test to measure reading comprehension and speed in university students.

**Method:**

The study was conducted under the quantitative approach, applied type, with non-experimental design. The type of sampling used was non-probabilistic and the sample consisted of 441 university students of both genders. The instrument prepared and applied was the Reading Speed and Reading Comprehension Test for Higher Education, which is an adaptation of the CompLEC test.

**Results:**

In the study, all the items had values above 0.80, which shows that the items were rated positively in terms of pertinence, relevance, and clarity. It was also evidenced that the model of three related factors presents adequate fit indices (CFI = 0.91; TLI = 0.90; RMSEA = 0.034 [IC90% 0.021 ‒ 0.046]; SRMR = 0.072). On the other hand, only the Recovery dimension (ordinal α = 0.62) presented acceptable reliability indices.

**Conclusion:**

The results show that the Reading Speed and Reading Comprehension Test for Higher Education has adequate psychometric characteristics in terms of content validity and internal structure of the scale. However, further studies are required to confirm the reliability of the scale.

## Introduction

Reading comprehension is one of the fundamental constructs in university education since it focuses on the traineeship of skills and competences for the production and acquisition of knowledge [1, 2]. For more than three decades, reading has been the subject of numerous studies in the international context. In Latin America, it has gained great relevance in the last decade, which has made it possible to identify some trends and models [3]. In Peru, reading comprehension continues to be a problem; it is one of the countries with the lowest performance in this competence [4]. Undoubtedly, being efficient in studying and reading is not only related to understanding what one reads, but also to the speed with which one reads. However, the latter has not received as much attention as reading comprehension, despite the fact that several studies show a high percentage of low reading speed [5]. In addition to this problem, unfortunately, in Latin America there is an evident need for a greater number of tests that measure reading speed and reading comprehension in university contexts.

With regard to the literature review, understanding a text is a process that involves constructing meanings from the reader’s prior knowledge of the information contained in the text [6, 7]. Likewise, understanding a text involves the use of cognitive and meta-cognitive processes that allow the reader to understand the meanings of the text read [8].

Moreover, reading comprehension is understood as a global process through the use of nine micro-skills: perception means training eye movement in order to increase reading speed and comprehension; memory allows the retention of information from the text read; anticipation allows the reader to hypothesise, predict events, facts or ideas; quick and attentive reading gives the reader the ability to move from one point to another in the text, identifying relevant data; inference gives the reader the ability to deduce implicit information from the text; main ideas allow the reader to identify the essential point, summary, communicative purpose of the author, etc.; structure and form, in this phase, the background and form of the text are analysed; reading between the lines refers to recognising the information that is not explicit; and self-assessment made of the reading process [9].

With regard to the reading competence, PISA 2018 defines it as “understanding, use, evaluation, reflection on and commitment with texts in order to achieve own goals, develop knowledge and personal potential, and participate in society” [10]. Under this definition, reading competence motivates cognitive, personal and social development, allowing the reader to actively participate in political, economic and cultural life with a critical and reflective stance for the construction of a country with quality of life. Text processing is carried out through three phases: locating relevant information, understanding the information and drawing inferences, and finally, evaluating and reflecting on the form and content of the text [10].

Reading competence involves a set of cognitive-linguistic skills that start from decoding to the elaboration of mental schemes that allow the comprehension of the text and the approach to knowledge [11]. With regard to reading levels, Pinzas [12] indicates three processes: Literal, inferential and critical comprehension. Literal comprehension: it means understanding the explicit information of the text, it is the first step for inferential and evaluative comprehension. At this level the reader identifies characters, place, main facts, ideas, causes and explicit characters in the text. Inferential comprehension: at this stage the reader infers implicit information, deduces meanings, makes conclusions, answers hypotheses, etc. Critical or evaluative comprehension: this is the highest level of comprehension; the reader makes value judgements about the form and content of the text, but this level is only reached when good literal and inferential comprehension has been achieved.

On the other hand, speed or quick reading began at the beginning of the 20th century, during the First World War; the strategists of the Air Force observed that most pilots could not distinguish planes at a certain distance, so they began to develop speed perception exercises with the tachistoscope, a machine that projected images for five hundredths of a second on a large screen, obtaining good results. This same method was applied in the field of reading, achieving a doubling of reading speed, but only for a few weeks. In the 1960s, Evelyn Wood, a school teacher, discovered that proper training of the eyes allowed quick movements, and that comprehension could be maintained above the 400 words per minute barrier [13].

Reading speed is defined as the number of words a person reads in a specific period of time, specifically minutes and seconds [14]. Speed reading allows for greater concentration, perceptual ability and overall comprehension of the text [15]. According to Horacio [16], the words read per minute is obtained by dividing the number of words read by the time spent. On the other hand, the words comprehended per minute is obtained by multiplying the words read per minute with the comprehension rate, and then dividing it by 100. The measure of reading speed and comprehension depends on the content of the text and other factors.

With regard to background, the studies by Inciso and García [17] conducted on 497 students from two universities in the city of Lima confirmed that the use of strategies to overcome reading comprehension problems favours the level of academic self-efficacy. Chuquichampi and Ricapa [18], for their part, developed an investigation with the in order to find out the influence of the application of the method from the Institute for Speed Reading, Studying and Memory *[Instituto de lectura veloz, estudio y memoria – ILVEM]* on the reading comprehension of 640 university students; the instruments used were readings and a questionnaire that was applied throughout the semester. One of the main findings were the significant mean differences in the pre- and post-test of words read per minute (PT 155, PsT = 277), comprehension rate (PT = 55%; PsT = 84%) and words comprehended per minute (PT = 90; PsT = 237). Therefore, it is summarised that the strategy employed using the ILVEM method influences the reading comprehension of students. The contributions of Cumpa and Cruz [19] allow us to affirm that the application of a comprehensive Reading Programme favours the development of reading comprehension in pre-university students. In Japan, Shimono [20] conducted a study with the purpose of comparing reading fluency in terms of speed and reading comprehension in groups of university students; the quasi-experimental design research involved the participation of 55 students divided into three groups, where the first group practised a combination of timed reading and repeated oral reading paying attention to fragmentation and prosody, while the second group was exclusively dedicated to timed reading, and the third comparison group was part of an oral communication training. As results, it was found that even though the three groups started with insignificant differences in reading comprehension according to the pre-test report. The post-test results confirmed an increase in the level of reading comprehension, demonstrating that timed and repeated oral reading practice is effective in increasing reading fluency, unlike a traditional curriculum.

Nevertheless, the findings of these studies suggest the presence of limiting factors in the achievement of reading comprehension and reading motivation. The study of Vázquez et al. [21], applied an instrument of 26 items grouped into four areas: reading habits, reading frequency, reading comprehension and reading experience. The findings show that students do not have motivation and habits to promote reading, especially due to their lack of interest and other factors. Finally, the contributions of Locher and Pfost, [22] resulting from the research carried out on 28,795 people including schoolchildren, university students and adults in Germany, with the objective to find a positive relationship between the time spent reading and reading comprehension skills, based on a questionnaire applied to find out how much time they spent reading, considering all reading opportunities, not just academic ones; likewise, they used tests consisting on five types of texts with four to eight questions. The results obtained in the university group showed that the correlation between reading while busy and reading in free time was very low (r = 0.16), and the relationship between reading comprehension related to study and reading in free time was also very low (r = 0.05). In conclusion, even though both variables are positively related, the low correlation could be due to qualitative changes in the habit of reading.

Therefore, the study of the constructs of reading speed and reading comprehension is of utmost importance because they play a fundamental role in the academic success of students at the higher education level, so it is necessary to have instruments that adequately measure these constructs. Therefore, this study is justified because it provides the academic community with a valid and reliable test to measure reading speed and reading comprehension in the university context.

Therefore, the general objective is to determine the psychometric properties of the revised compLEC test to measure reading comprehension and speed in university students. Likewise, the specific objectives proposed are: to determine the validity based on the internal structure through techniques based on the Classical Test Theory and the Item Response Theory, to determine the factorial invariance of the scale according to the gender and age of the participants, and to determine the reliability of the scale.

## Methodology

### Design

The method used was hypothetical-deductive, which is characterised by deductive inferences that are tested until conclusions are reached [23]. The study has a quantitative approach whose purpose is the measurement and quantification of its variables to test hypotheses and build theories [24]. It was an applied research which has the purpose of applying existing scientific knowledge in order to know, understand, act and modify a given reality [23]. The study has a non-experimental design because the study variables will not be manipulated [25]. The population consisted of the students of the General Studies Programme. The technique used was the survey.

### Participants

Data collection was made through non-probability sampling, using the following inclusion criteria: (a) informed consent of participants, (b) ability to read and write in Spanish, (c) enrolment in a university course of study. The sample consisted of 441 undergraduate students of both genders (30.2% male and 69.8% female) between the ages of 16 and 62 years (M = 23.4; SD = 8.4). The students were in the first year under the regular modality and career change. They were also taking the subject of Writing academic texts. With regard to the career studies, a percentage of university students studied medicine (20%), nursing (17.2%), dentistry (15.6%) and pharmacy (10.4%). In addition, most of the participants live in urban areas (84.1%) and very few in rural areas (15.9%). Finally, even though the majority of university students indicate that they do not work (51.5%), there is an important group that does work and study (48.5%).

### Instrument

#### Reading speed and reading comprehension test for higher education

The Reading Literacy Test for Secondary Education (CompLEC) was elaborated considering the theories proposed by the PISA-2000 report, and is composed of five texts with 20 questions. Three continuous texts: one argumentative: *Nuclear energy*; two expository: *The language of bees* and *Sit in the right chairs*. The discontinuous texts are two: *Global warming and Traffic accidents.* CompLEC was scaled with a sample of 1,854 students, and the results showed that it has satisfactory reliability, homogeneity and validity properties [26].

To prepare the *Reading Speed and Reading Comprehension Test for Higher Education*, the CompLEC test was adapted, considering only continuous texts; five questions correspond to the Information Retrieval dimension, seven questions to the Integration and Interpretation of Information dimension and three questions to the Reflection and Evaluation of Information dimension. It should be noted that two questions from the Reflection and evaluation of information dimension were added to the text *Sit in the right chairs*: 14) Why is the phrase: “Prevention is better than cure” mentioned? and 15) The text says: “(…) even though it may be more expensive, over time the benefits outweigh the initial cost”? Why does the text include this information? (See Table 1).

**Table 1:** Measurement of the variable reading comprehension

Dimension	Indicator	Items	Scales and values	Levels and Range by Dimension	Levels and Range - General
Retrieval of information	• Identifies isolated data in the text. • Identifies specific data in the text.	05 (2, 4, 6, 9, 11)	Interval scale 0 = incorrect 5 = correct	Very high (87 − 100%) High (75 − 86%) Medium (61 − 74%) Low (50 − 60%) Very low (Less than 50%)	Very high: 66–75 (87 − 100%) High: 56–65 (75 − 86%) Medium: 46–55 (61 − 74%) Low: 36–45 (50 − 60%) Very low: 0–35 (Less than 50%)
Integration and interpretation of information	• Summarises messages. • Relates ideas. • Prepares inferences	07 (1, 5, 7, 8, 10, 12, 13)			
Reflection and evaluation of information	• Evaluates the content of the text. • Evaluates the relevance of the text form	03 (3,14, 15)			

With regard to the reading speed variable, the instrument consists of three readings, each with a defined number of words to determine the speed levels of the reader, divided into slow, normal and fast (See Table 2).

**Table 2:** Measurement of the variable reading speed

Dimension	Indicator	No. of words per text	Scale	Levels and range - General
Reading speed	Words read per minute (W.P.M.)	1. The language of bees (426 words) 2. Nuclear energy (368 words) 3. Sit in the right chairs (275 words)	Slow Normal Fast	Slow (WPM = 200) Normal (WPM + 300) Fast (WPM + 400)

#### Procedure

In order to conduct the study, we obtained the authorisation from the Institutional Ethics Committee of the University Norbert Wiener with Registration No.2265 − 2022, where the research was conducted and we complied with the requirements of the Declaration of Helsinki [27]. Initially, content validity was carried out by five judges, who certified the pertinence, coherence and clarity of the content in the Peruvian context. The test was applied online, the software automatically marked the time that the student spent on each text; likewise, it automatically scored the number of correct answers per question in the three texts. Socio-demographic data were collected through a form using the Google Forms platform. In the first section of the form, informed consent was obtained from the participants of the study; it was explained about the anonymity and that the data obtained are confidential. The application of the form was carried out through the virtual board and only to students who marked the agreement option.

### Data analysis

In the study, the Diagonally Weighted Least Squares with Mean and Variance corrected (WLSMV) estimator was used to perform the Confirmatory Factor Analysis (CFA). The RMSEA, SRMR, CFI and TLI indices were used to assess model fit. For the RMSEA and SRMR indices, values below 0.08 were considered acceptable [28]. For the CFI and TLI indices, values greater than 0.95 were considered adequate [29]. The reliability of the scale was assessed through Cronbach’s alpha coefficient [30] and the omega coefficient [31]. Regarding the item analysis, the following levels of difficulty were used: < 0.20 (very difficult); 0.20 − 0.39 (difficult); 0.40 − 0.59 (moderately difficult); 0.60 − 0.79 (moderately easy), 0.80 − 0.89 (easy) and ≥ 0.90 (very easy). For the discrimination indices, the following levels of interpretation were used: < 0.20 (low); 0.20 − 0.29 (moderate); 0.30 − 0.39 (high) and ≥ 0.40 (very high).

For the statistical analysis, the RStudio environment [32] for RCore Team [33] was used. Specifically, the “lavaan” package [34] was used to perform the CFA and the “sjPlot” package was used to evaluate the discrimination and difficulty indices [35].

## Results

### Content validity

In the study, all the items had values above 0.80, which shows that the items were rated positively in terms of relevance, pertinence and clarity, with values close to 1, indicating that the judges agreed. Furthermore, these values show that the items are related and fundamental to the assessment of the construct, containing easily understood vocabulary that fits the context.

### Descriptive analysis of the items

Table 3 shows that in the Retrieval dimension, item 6 has the highest average score among university students. In the Integration dimension, item 8 has the highest average score. For the Reflection dimension, item 14 has the highest average score. On the other hand, it is observed in Table 1 that all the items present values of asymmetry and kurtosis within the expected limits (As < ± 2; Ku < ± 7), according to the criteria of Finney and DiStefano [36]. (See Table 3)

**Table 3:** Descriptive analysis of the items

Dimension	Items	*M*	*SD*	*g*1	*g*2	*p*	*d*
Retrieval	2.- A scout bee has found food in the opposite direction to the sun. How will the bee indicate to its companions the location of the food?	0.62	0.49	− 0.48	-1.78	0.62	0.34
	4.- ¿What is the shape of the bees dance when the food source is 30 m away from the hive?	0.49	0.50	0.04	-2.01	0.49	0.34
	6.- For Arturo, nuclear energy is the best alternative. Why?	0.68	0.47	− 0.78	-1.4	0.68	0.27
	9.- Luis is an engineer who has many years working in a nuclear power station and thinks that the work he performs is an important contribution to society. With whom do you think he would agree (with Arturo or with Sonia)?	0.52	0.50	− 0.10	− 0.20	0.52	0.22
	11.- Which of the following statements is associated with the characteristics that a suitable chair should have?	0.34	0.47	0.69	-1.53	0.34	0.20
Integration	1.- The purpose of the section entitled A mutual benefit is to explain:	0.60	0.49	− 0.41	-1.84	0.60	0.33
	5.- Bees transport pollen from a flower to another flower taking the pollen:	0.42	0.49	0.34	-1.9	0.42	0.18
	7.- Even though there are many disagreement points, Arturo and Sonia agree that …	0.49	0.50	0.05	-2.01	0.49	0.21
	8.- Arturo suggests that the use of nuclear power could, in part, avoid climate change because …	0.64	0.48	− 0.60	-1.64	0.64	0.27
	10.- For what purpose do Arturo and Sonia make reference to nuclear accidents?	0.53	0.50	− 0.13	-1.99	0.53	0.26
	12.- Why is sitting inadequately more harmful at work than sitting in chairs at home?	0.57	0.50	− 0.28	-1.93	0.57	0.34
	13.- Diseases caused by Repetitive Strain Injuries (SRI) are characterised by:	0.61	0.49	− 0.45	-1.8	0.61	0.14
Reflection	3.- Why is it said that bees have language?	0.48	0.50	0.09	− 0.20	0.48	0.20
	14.- Why is the phrase: “Prevention is better than cure” mentioned?	0.58	0.49	− 0.33	-1.9	0.58	0.25
	15.- The text says: “(…) even though it may be more expensive, over time the benefits outweigh the initial cost” Why does the text include this information?	0.45	0.50	0.19	-1.97	0.45	0.15

*Note*. *M* = Mean; *SD* = Standard Deviation; *g1* = Skewness; *g2* = Kurtosis; *p* = Difficulty index; *d* = Discrimination index

### Discrimination analysis and item difficulty

Table 3 shows that all the items of the scale present adequate difficulty indices, where item 11 is the most difficult question of the scale. In addition, items 4, 9, 5, 7, 10, 12, 3, 14 and 15 are moderately difficult questions as they are within the range of 0.40 to 0.59. It is also observed that items 2, 6, 1, 8 and 13 are moderately easy questions as they are within the range of 0.60 to 0.79. Therefore, all the items of the scale present adequate difficulty indices, which allows for an adequate assessment of the construct.

With respect to the discrimination indices, it can be seen in Table 1 that items 6, 9, 11, 7, 8, 10, 3 and 14 show moderate levels of discrimination as they are within the range of 0.20 to 0.29. In addition, items 2, 4, 1 and 12 show adequate levels of discrimination, ranging from 0.30 to 0.39. However, items 5, 13 and 15 show values below 0.20, which is evidence of low levels of discrimination. This shows that most of the items of the scale present adequate levels of discrimination, which allows us to properly distinguish students with high and low levels of the construct.

### Validity based on internal structure

The study found that the original three-factor related model presented adequate fit indices (χ2 = 130.29; df = 86; *p* < 0.01; CFI = 0.91; TLI = 0.90; RMSEA = 0.034 [90%CI 0.021 − 0.046]; SRMR = 0.072). Furthermore, it can be seen in Table 4 that most of the items have moderate factor loadings in their corresponding factor. However, in the Integration dimension, items 7 (λ = 0.27) and 12 (λ = 0.23) have very low factor loadings. Similarly, in the Reflection dimension, item 15 (λ = 0.23) shows a very low factor loading (see Table 4).

**Table 4:** Confirmatory factor analysis of the revised CompLEC scale

Items/Factors	CompLEC-R		
	F1	F2	F3
2	0.56		
4	0.30		
6	0.53		
9	0.47		
11	0.58		
1		0.61	
5		0.37	
7		0.27	
8		0.35	
10		0.45	
12		0.23	
13		0.33	
3			0.49
14			0.41
15			0.23
F1	‒	0.92	0.96
F2		‒	0.80
F3			‒

### Reliability of the scale

In the total study sample, the scale has adequate reliability indices (ordinal α = 0.73). Regarding the dimensions of the instrument, only the Recovery dimension presented acceptable internal consistency indices (ordinal α = 0.62). The Integration (ordinal α = 0.50) and Reflection (ordinal α = 0.36) dimensions presented low levels of internal consistency.

### CompLEC-R test results

Table 5 shows that only 15.4% of the participants answered the five questions on the recovery dimension correctly. On the other hand, 24% managed to answer only two questions of this dimension. Regarding the integration dimension, only some students could correctly answer the seven questions of this dimension (2.5%). 22.4% and 21.3% of students answered three and four questions correctly, respectively. Finally, in the reflection dimension, only 23.1% answered the three questions belonging to this dimension correctly. 33.6% of the students could only correctly answer one question of this dimension.

**Table 5:** *CompLEC test result*

Dimensions	Number of correct answers							
	0	1	2	3	4	5	6	7
Retrieval	3.6%	16.1%	24%	21.8%	19%	15.4%	‒	‒
Integration	2%	9.1%	17.2%	22.4%	21.3%	17.9%	7.5%	2.5%
Reflection	9.1%	33.6%	34.2%	23.1%	‒	‒	‒	‒

## Discussion

The study has focused on demonstrating the validity, descriptive and discriminant analysis of the items of two instruments to measure reading speed and reading comprehension in university students. For the reliability of the scale, Cronbach’s alpha and the omega coefficient were used, which yielded values of 0.73 and 0.63. In this respect, the validity and reliability of an instrument is part of a process to verify its consistency in the measurement of a variable, taking into account the purpose and the context where it will be applied [37] y [38].

About this, although the overall reliability of the scale shows adequate internal consistency indices, the dimensions present low-reliability values; this could be associated with the low factor weights of some items in the dimensions, especially in the reflection factor. Therefore, interpreting the scale scores at the dimension level would be imprecise and with a high degree of error. Against this, the test’s total score could be used since it shows better reliability indices. This procedure could be supported by the degree of relationship between the dimensions, which is very high.

With regard to the analysis of the items, based on the levels of difficulty: very difficult, difficult, moderately difficult, moderately easy, easy, very easy. It can be affirmed from the findings that the items of the instruments have an adequate level of difficulty. For Hurtado [39], this means that the difficulty index is the degree of difficulty of a question to be answered correctly in the sample under study. On the other hand, the discrimination indexes used the following levels of interpretation: low, moderate, high and very high. In this sense, it can be affirmed that the results position the items with adequate discrimination values. For the same author, a question that discriminates adequately in an instrument is because it allows differences to be established between individuals with higher and lower scores, as well as ensuring the existence of a positive correlation between the scores of the items and the instrument. However, there are items that have low discrimination indices such as 7 (Even though there are many disagreement points, Arturo and Sonia agree that…) and 15 (The text says: “(…) even though it may be more expensive, over time the benefits outweigh the initial cost” Why does the text include this information?), integration and reflection questions respectively, which requires an inferential and critical analysis of the text so that a value judgment can be made and alternative solutions can be proposed. considered as high levels of reading comprehension. On the other hand, the evaluation was subject to an established time to develop the reading comprehension questions, which could have been a factor in the low discrimination index.

Reading speed is determined by the number of words read per minute; in the study it was established as a scale of measurement that = 200 words have a slow level, + 300 words have a normal level and + 400 have a fast level of reading. And for the reading comprehension variable with its three dimensions: retrieval of information, integration and interpretation of information, and reflection and evaluation, the following scale of measurement was established: very high (87–100%), high (75–86%), medium (61–74%), low (50–60%) and very low (less than 50%). In the quasi-experimental study by Chuquichampi and Ricapa [18], after applying the ILVEM method to 640 university students, they used 1350 word readings as a measurement instrument. The post-test result reported that an average of 237 words were read per minute, placing the students who participated in the research on the borderline of a slow to normal level. Also, the comprehension rate reached 84% [18], placing the group at a high level. One of the limitations of the study is that data collection was made through judgement non-probabilistic sampling, the disadvantage of which is that it does not allow for generalisation of the results. In this sense, it is suggested that future studies use probability sampling. Another limitation to mention is that the participants were from the area of health sciences, which limited the evaluation of the construct in other areas such as engineering, social sciences and humanities. It is suggested that future studies expand the sample size for a better understanding of the construct. Another limitation of the study was that competing models were not tested for the factorial structure of the scale, such as one-dimensional, Bi-factor, and ESEM models, since the relationship between the dimensions was very high. Therefore, it is suggested to test these models with larger and more representative population samples. Finally, another limitation found is that the construct was analysed only for Peruvian university students. Therefore, cross-cultural studies are suggested to study whether the construct is similar in other Latin American countries. However, the limitations mentioned should be addressed in future research to enhance the generalizability and applicability of the findings.

## Conclusion

The results show that the Reading Speed and Reading Comprehension Test for Higher Education presents adequate psychometric characteristics regarding content validity, internal structure and reliability of the global scale. However, the instrument showed low levels of reliability in the three dimensions evaluated. Consequently, this instrument requires more evidence of its psychometric performance, especially on the reliability of the dimensions. Despite this, the present study shows the first psychometric evidence of the revised CompLEC test in Spanish. These results are important because they will allow researchers and educators to collect information for decision-making based on the results obtained. Knowing the mastery of these variables in university students will allow them to plan and apply projects, modules, strategies, and techniques that contribute to developing their reading competence and speed, which are essential for acquiring knowledge and strengthening their research skills and professional training.

## Data Availability

The datasets used and/or analysed during the current study available from the corresponding author on reasonable request.
